# Big Data, artificial intelligence and laboratory medicine: time for integration

**DOI:** 10.1515/almed-2021-0003

**Published:** 2021-02-10

**Authors:** Damien Gruson

**Affiliations:** Pôle de recherche en Endocrinologie, Diabète et Nutrition, Institut de Recherche Expérimentale et Clinique, Cliniques Universitaires St-Luc and Université Catholique de Louvain, Brussels, Belgium; Department of Clinical Biochemistry, Cliniques Universitaires St-Luc and Université Catholique de Louvain, Brussels, Belgium

**Keywords:** artificial intelligence, Big Data, cancer, cardiovascular, ethical, machine learning

Laboratory medicine is continuously integrating innovation to support clinical decision, disease monitoring and patient safety [1]. Innovation has an enormous potential to transform healthcare systems and laboratory medicine, and can provide to healthcare workforces the insight and tools to offer higher quality of care to more patients as well as improving health outcomes with fewer resources [2]. With the current innovation comes also the power of data and Artificial intelligence (AI). Data science and AI are already revolutionizing our citizen and daily life and the way institutions, cities, exchanges and businesses operate. AI is a broad term that combines computation with sophisticated mathematical models and in turn allows the development of complex algorithms which are capable to simulate human intelligence such as problem solving and learning [2]. One of the most promising fields of applications of Big Data and AI is healthcare, where AI has the potential to revolutionize existing protocols for diagnostics as well as disease prevention and control, significantly boosting patient safety and quality of care [3]. Because of a continuously increasing accessibility to high volumes of data (through electronic medical records, laboratory informatic systems, omics, digital applications…), the promises and expectations related to the fields of Big Data and AI are growing exponentially.

One recent accelerator for this growth is certainly the COVID-19 outbreak. If the sanitary and human consequences of the COVID-19 over the world are terrible, the pandemic plays a role as a catalyst for innovation and AI. The principle of adaptation to the virus drive to a changing technological environment and overall existing practices in order to meet ever growing demand for quality care in the conditions of limited resource availability [2]. Recently, an Opinion of the Expert Panel on Effective Ways of Investing in Health of the European Commission on the organization of resilient health and social care following the COVID-19 pandemic was published [4]. The opinion identifies the building blocks of resilient health and social care, explores the elements and conditions for capacity building to strengthen health system resilience, addresses healthcare needs of vulnerable patients at times of crisis, and defines a blueprint for resilience testing of health systems. Among the different elements discussed, the power of data and value of data integration and AI to face unexpected outbreaks was emphasized [4]. Several examples of applications of AI to COVID-19 are already reported such as AI-enabled outbreak tracking apps, chatbots for diagnostics, AI-powered analysis of scientific publications and triage using natural language processing for screening potential patients and prognosis prediction tools, using radiology CT scans to manage system capacities [2].

Multiple examples of evidence and added value of Big Data and AI are also now available for the management of non-communicable chronic diseases such as cancer or cardiovascular disorders. The aim of AI tools is augmenting the effectiveness of the physicians and accuracy of clinical decision as well as extending better quality to patients and improving patient safety. Big Data and AI have clearly the potential to support decision making by improving both diagnostic and prognostic performances. In the fields of cancer and cardiovascular medicine, AI has already proven promising in the extraction of deep phenotypic information from imaging and laboratory data, and other medical devices [2], [ 3]. AI has clearly value to explore novel genotypes and phenotypes in existing chronic diseases, to improve the quality of patient care and to enable cost-effectiveness with reduce readmission and mortality rates [5]. AI-based technologies can also guide treatment and its potential toxicity as well as predict the likelihood of failure of clinical trials and side effects of polypharmacy combinations. Furthermore, highly sophisticated AI-powered tools such as the use of “digital twins” can allow physicians to anticipate the response of a certain illness or drug in patients and assist clinical teams in the preparation of clinical trials by matching suitable patients to appropriate trials [2].

Big Data and AI appear clearly as enabler for personalised medicine through early risk prediction, prevention and therapeutic intervention. Laboratory and biological data will markedly contribute to the efficiency and quality of the AI tools. Beside the clinical impact, it is also important to mention the power of Big Data and AI to improve laboratory efficiency and sustainability by identifying area of waste, improving processes and allowing a more rational laboratory test ordering [6].

As the value and benefits of Big Data and AI are concrete as ever the importance of a successful integration to practices is critical. However, many challenges still remain and are listed in Table 1 [2]. Priorities have to be placed on the building of data ecosystems and infrastructures which will fuel and shape AI, integrate and took advantage of the future “European Data Space”, engage healthcare workforces in external validation and proving generalizability of AI applications, incorporate digital and AI-related content into the current education and training frameworks for healthcare professionals, develop a robust regulatory and ethical framework based on a patient-centric risk-based approach to AI [2], [ 5]. Specialists in laboratory medicine and clinical laboratories will be key players of this evolution and their missions will at least include providing structured and standardized data, counselling on the type of data to be used, integrate multidisciplinary teams for the validation of the AI derived tools and use the tolls for optimization of patient safety, laboratory processes and workflows.

**Table 1:** Challenges around big data and AI [2].

1.	Establish a comprehensive legal framework for AI and update existing relevant legislations in order to ensure that they are fit for purpose.
2.	Identify and promote best practices ensuring the robustness of big data and AI systems in the health sector both at the stages of development and actual use to reduce potential biases and errors of AI-based decision making.
3.	Accelerate the development of a common European Health data space as part of a comprehensive strategy to address the current fragmentation of the EU health data landscape.
4.	Improve data interoperability and support the development of data infrastructure, with the goal of providing a reliable flow data with standardized formats, the necessary cybersecurity provisions and data protections
5.	Support the development of national electronic health records and improve the interoperability of health data.
6.	Equip the workforce with the necessary skill sets to maximize the positive impact of AI and conduct a comprehensive regulatory assessment of the medical professions frameworks to determine whether they are fit for the use of patient-centred AI in healthcare provision.
7.	Ensure that AI is applied in full respect of EU data protection rules while observing the balance between scientific advancement and patient protection.
8.	Invest in research and innovation to boost the updates of AI applications to healthcare and support patient access to the best available technologies.
9.	Put in place mechanisms to ensure educational assistance to patients to allow them to better understand and use AI and empower them to actively participate in the management of their health.

In conclusion, the use of data science Big Data and AI Artificial intelligence in their various applications can truly overhaul patients’ healthcare experience throughout the entire healthcare pathway from prevention, to screening, to early diagnosis to treatment and disease management. Specialists in laboratory medicine and clinical laboratories will be key players in the efficient and safe integration of Big Data and AI tools as well as in the training of workforce and education of patients. With such a careful integration, Big Data and AI-technologies can have significant socioeconomic impact on European healthcare systems.
